# Assessing the contribution of thrombospondin-4 induction and ATF6α activation to endoplasmic reticulum expansion and phenotypic modulation in bladder outlet obstruction

**DOI:** 10.1038/srep32449

**Published:** 2016-09-01

**Authors:** Katarzyna K. Krawczyk, Mari Ekman, Catarina Rippe, Mario Grossi, Bengt-Olof Nilsson, Sebastian Albinsson, Bengt Uvelius, Karl Swärd

**Affiliations:** 1 Department of Experimental Medical Science, Lund University, Lund, Sweden; 2 Department of Urology, Clinical Sciences, Lund University, Lund, Sweden

## Abstract

Phenotypic modulation of smooth muscle cells is a hallmark of disease. The associated expansion of endoplasmic reticulum (ER) volume remains unexplained. Thrombospondin-4 was recently found to promote ATF6α activation leading to ER expansion. Using bladder outlet obstruction as a paradigm for phenotypic modulation, we tested if thrombospondin-4 is induced in association with ATF6α activation and ER expansion. Thrombospondin-4 was induced and ATF6α was activated after outlet obstruction in rodents. Increased abundance of spliced of Xbp1, another ER-stress sensor, and induction of Atf4 and Creb3l2 was also seen. Downstream of ATF6α, Calr, Manf, Sdf2l1 and Pdi increased as did ER size, whereas contractile markers were reduced. Overexpression of ATF6α, but not of thrombospondin-4, increased Calr, Manf, Sdf2l1 and Pdi and caused ER expansion, but the contractile markers were inert. Knockout of thrombospondin-4 neither affected bladder growth nor expression of ATF6α target genes, and repression of contractile markers was the same, even if ATF6α activation was curtailed. Increases of Xbp1s, Atf4 and Creb3l2 were similar. Our findings demonstrate reciprocal regulation of the unfolded protein response, including ATF6α activation and ER expansion, and reduced contractile differentiation in bladder outlet obstruction occurring independently of thrombospondin-4, which however is a sensitive indicator of obstruction.

Smooth muscle cells (SMCs) change their properties in response to physiological and pathological cues^1^,^2^,^3^,^4^. For instance, SMCs in the media of healthy arteries are in a contractile state characterized by a low rate of proliferation and a high expression of myofilament proteins, allowing them to shorten and thereby regulate arterial diameter. Following injury, a change in phenotype occurs and SMCs become proliferative and increase their synthesis of matrix molecules. The earliest description of this phenomenon, based on observations of visceral SMCs in primary culture, dates more than 50 years back^3^,^5^. The transition to the synthetic phenotype involves a reduction of myofilaments and expansion of rough endoplasmic reticulum and Golgi^2^,^3^,^6^. Phenotypic modulation is considered to play roles in a wide range of pathological conditions, including arterial lesions^1^,^7^,^8^, bladder outlet obstruction^9^, and following mechanical distension of the intestine^2^.

Myocardin and the myocardin related transcriptional coactivators are critical drivers of the contractile SMC phenotype^8^,^10^. A key concept in phenotypic modulation is competition between myocardin and ternary complex factors (TCFs), such as Elk-1, for binding to serum response factor (SRF)^11^, which mediates many of the effects of these coactivators on transcription. Myocardin and Elk-1 bind to a common site on SRF in a mutually exclusive manner. The myocardin/SRF complex drives transcription of SMC differentiation markers including myosin heavy chain, smooth muscle α-actin, calponin and SM22α^8^,^12^. The Elk-1/SRF complex, on the other hand, drives expression of growth factor-responsive genes such as c-Fos^13^. Many studies have elaborated further on this paradigm of phenotypic modulation^14^,^15^, but no current model explains the expansion of the endoplasmic reticulum (ER) that occurs as SMCs assume the synthetic phenotype.

ATF6α (*Atf6*) is a basic leucine zipper protein of 90 kDa in the ER membrane^16^,^17^. In response to ER stress, such as when the demand on ER protein synthesis and folding increases, it traffics to the Golgi where it is cleaved by Site 1 and Site 2 proteases giving rise to a ≈50 kDa protein (ATF6α-P50) that enters the nucleus. ATF6α-P50 binds to ER stress elements in the genome^17^, and drives transcription of genes of relevance for ER proteostasis. ATF6α is one of three arms of the unfolded protein response (UPR), the others being IRE1/XBP1 and PERK/IEF2α. Target genes of ATF6α include those that facilitate protein folding, those that resolve inappropriately formed disulfide bonds, and those that cause degradation of misfolded proteins^18^. ATF6α also expands the ER volume and the secretory pathway, in part by increasing the biosynthesis of phosphatidylcholine and phosphatidyl-ethanolamine, and by increasing the ratio of free to esterified cholesterol^19^. Prior work demonstrated that binding of ATF6α to SRF is important for serum induction of c-Fos^20^, and recent findings show that ATF6α may drive expression of myocardin in differentiation of stem cells towards the SMC lineage^21^. These observations raise the possibility that ATF6α may be responsible for certain aspects of phenotypic modulation, including ER expansion, upon switching of SMCs to the synthetic phenotype.

Until recently, the molecular understanding of steps leading to ATF6α cleavage in the Golgi apparatus has lagged behind understanding of how other arms of the unfolded protein response are activated^22^. Work in the heart demonstrated that the stress-inducible protein thrombospondin-4 (Thbs4) binds to the ER luminal domain of ATF6α and facilitates its cleavage^23^. These findings argued for a role of thrombospondin-4 as a muscle-specific effector of the UPR leading to ER expansion^23^. Of interest in regard to phenotypic modulation of SMCs is the prior demonstration that Thbs4 increases considerably in the aorta and in small mesenteric arteries in hypertension^24^,^25^.

The aim of the present study was to test if Thbs4/ATF6α signaling is activated during phenotypic modulation of SMCs and to examine whether this associates with ER expansion. We used an *in vivo* model of partial bladder outlet obstruction^26^ which in rat leads to a 10-fold increase of bladder weight over a 6-week period. The bladder growth in this model is highest over the first 10 days and ERK1/2 activation peaks on day 4^27^. Studies have demonstrated that outlet obstruction leads to time-dependent changes in bladder contractility^26^,^28^, and accompanying changes in the expression of contractile proteins^9^,^29^,^30^, typical for the switch from contractile to synthetic phenotype. Whether bladder outlet obstruction associates with ER expansion is not known, but in all other regards this model seems suitable for studies of phenotypic modulation of visceral smooth muscle. Here we demonstrate reciprocal regulation of ATF6α targets and contractile markers in bladder SMCs occurring independently of Thbs4.

## Results

### Thbs4 accumulates intracellularly in detrusor myocytes following bladder outlet obstruction

**Figure 1:**
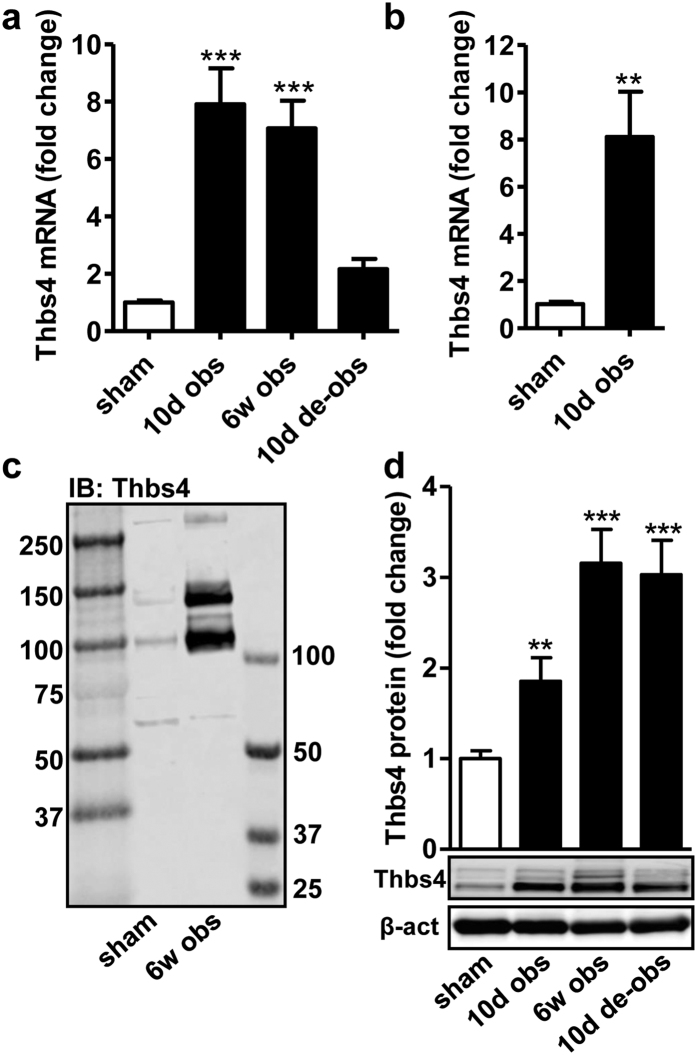
Thrombospondin-4 (Thbs4) is induced at the mRNA and protein level in the rat bladder following urethral obstruction. Rats were either sham-operated or obstructed for 10 days and 6 weeks, respectively. In one group of rats the obstruction was relieved for 10 days following 6 weeks of obstruction. Analysis of gene activity by microarray analysis (GSE47080, n = 6–8 at all times) demonstrated sizeable induction of Thbs4 (panel a). Independent confirmation by qRT-PCR of the up-regulation of Thbs4 is shown in panel b. Panel c shows immunoblotting (IB) for the Thbs4 protein in control and obstructed bladders. Two different size standards were used. Panel d shows summarized data on the Thbs4 protein at different times of obstruction. Means ± S.E.M. of 6–8 independent replicates are depicted in this and the following figures.

Global analysis of gene activity in microarray experiments (GEO accession number GSE47080) disclosed impressive induction of Thbs4 mRNA in the bladder at 10 days and at 6 weeks of obstruction followed by a return towards control level on de-obstruction (Fig. 1a). In view of recent evidence that Thbs4 is upstream of ATF6α leading to endoplasmic reticulum (ER) expansion in the heart^23^, we set out to confirm Thbs4 induction and to examine its association with ATF6α-driven gene expression. First, the up-regulation of Thbs4 mRNA at 10 days was confirmed using qRT-PCR (Fig. 1b). Using western blotting we found two major Thbs4 bands migrating between 100 and 150 kDa on immunoblotting of sham-operated and obstructed bladders using two different antibodies (Fig. 1c and data not shown); both increased dramatically following obstruction. This is in agreement with previous work showing that rat Thbs4 migrates as two bands between 100 and 150 kDa^31^. Thbs4 was maintained at an elevated level for at least 10 days following de-obstruction (Fig. 1d), despite a decline of the mRNA level, suggesting slow Thbs4 protein turnover.

**Figure 2:**
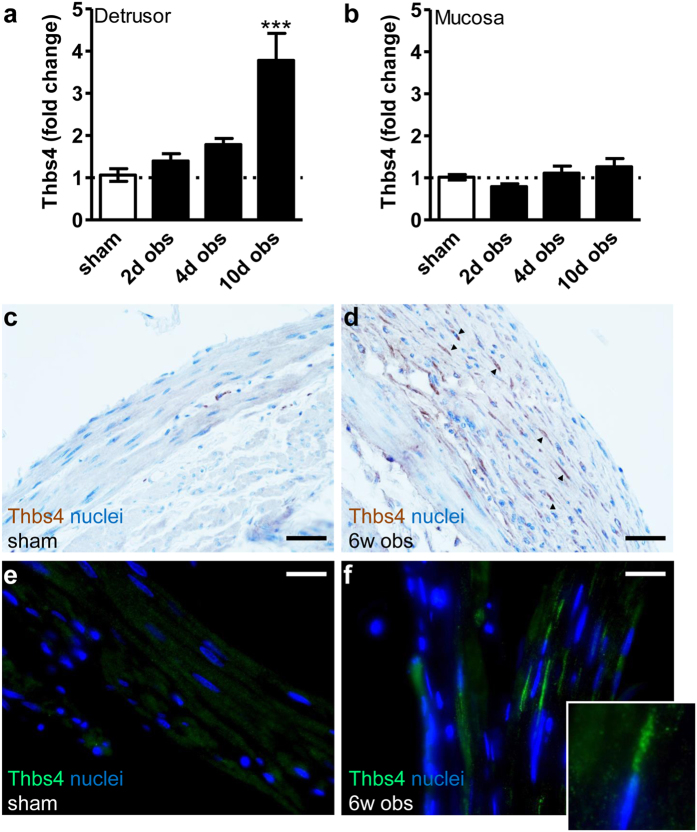
Thbs4 induction is specific for the detrusor layer and does not occur in the mucosa. Rats were sham-operated or obstructed for 2, 4, and 10 days. Following bladder excision, the detrusor and mucosal layers were separated by microdissection and RNA was isolated. qRT-PCR showed significant Thbs4 induction in the detrusor (**a**) but no change in the mucosa (**b**). Immunohistochemistry (DAB, brown, panels c and d) for Thbs4 confirmed increased staining as well as thickening of the detrusor layer after 6 weeks of obstruction. Thbs4 positive cells are indicated by black arrowheads in panel d. Examination of detrusor at higher magnification using immunofluorescence revealed that Thbs4 (green) accumulated intracellularly close to the nuclear poles (panels e,f). Black and white scale bars represent 200 and 100 μm, respectively. The inset in (**f**) shows one example of a Thbs4 positive cell where Thbs4 staining (green) radiates out from the nucleus (blue).

To examine in which cell types Thbs4 increases, we obstructed another series of rats and isolated the mucosal and detrusor layers by micro-dissection. Thbs4 mRNA induction was specific for the detrusor and was not seen in the mucosa (Fig. 2a,b). Immunohistochemical staining for Thbs4 showed that the detrusor from sham-operated rats was largely Thbs4-negative and that a sizeable fraction of SMCs stained positive for Thbs4 following obstruction (brown in Fig. 2c,d). Immunofluorescence (Fig. 2e,f) confirmed increased abundance of Thbs4 positive cells (green) following obstruction and demonstrated that staining was restricted to intracellular structures radiating from SMC nuclei (inset in Fig. 2f). Such an intracellular localization is a prerequisite for ATF6α activation by Thbs4^23^.

### Bladder outlet obstruction activates ATF6α and increases ATF6α targets in both rats and mice

**Figure 3:**
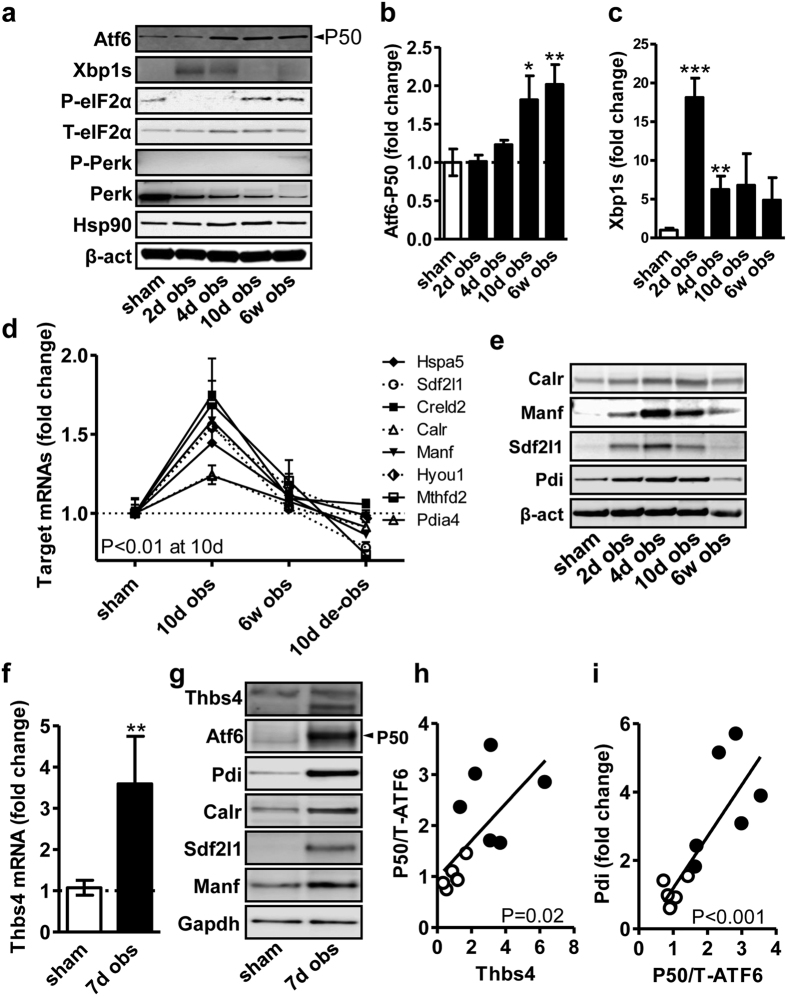
ATF6α is activated and ATF6α target genes are transcribed in both rat and mouse bladders following outlet obstruction. The 50 kDa cleaved and active form of ATF6α, spliced XBP1 (Xbp1s, 54 kDa) and phosphorylated Perk/eIF2α, three arms of the classical ER stress pathway, were assayed by western blotting at different times of outlet obstruction (**a**). Summarized data on ATF6α-P50 is shown in panel b and summarized data on the spliced form of XBP1 is shown in (**c**). Panel d shows time courses of ATF6α target genes from the microarray experiment (GSE47080, the P-value is for comparison of 10 d vs. sham). Panel e shows protein levels of ATF6α targets in a new experiment (different set of bladders than those in Fig. 3a). Panel f shows that Thbs4 mRNA is induced in the mouse bladder following 7 days of outlet obstruction. Panel g shows a stack of blots for Thbs4, ATF6α and ATF6α target genes in control and obstructed mouse bladders. Panel h and i show correlation analyses for Thbs4 versus cleaved (50 kDa) over total ATF6α and for cleaved over total ATF6α versus Pdi, respectively. P-values from Spearmann correlation analyses are given in the panels.

We next assayed the ≈50 kDa cleaved and active form of ATF6α using western blotting. ATF6α-P50 increased at 10 days and at 6 weeks of outlet obstruction (Fig. 3a,b). This was confirmed using another ATF6α antibody (Supplemental Fig. 1a through d). To support ATF6α activation beyond its cleavage we used transcription factor binding site (TFBS) analysis. This is a method to measure enrichment of DNA motifs among promoters of differentially expressed genes in microarray experiments. Significant (P = 0.003) enrichment of ATF6α motifs was found at 10 days of obstruction. Similar results were obtained when we compared mean fold changes of ATF6α targets^16^ in the array data using a Wilcoxon signed rank test (Supplemental Fig. 1e,f). Figure 3d shows time-courses for eight Thbs4-responsive and ATF6α-dependent^23^ mRNA targets in outlet obstruction. Using a new series of obstructed rats we next examined the ATF6α targets calreticulin (Calr), mesencephalic astrocyte-derived neurotrophic factor (Manf, also known as Armet), stromal cell-derived factor 2-like 1 (Sdf2l1) and protein disulfide isomerase (Pdi) at the protein level and found them to be increased with a peak at 4–10 days (Fig. 3e). In summary, our findings so far provided support for Thbs4 accumulation, ATF6α cleavage and downstream ATF6α-dependent transcriptional activation in rat bladder outlet obstruction. TFBS analysis did not support activation of XBP1 (P = 0.13), a second arm of the unfolded protein response, but the spliced (54 kDa) form of XBP1 (XBP1s) increased transiently as shown using western blotting (Fig. 3a,c), and target genes specific for XBP1 were accordingly increased (Supplemental Fig. 2a). Phosphorylation (at Ser51) of eIF2α, representing a third signaling arm of the unfolded protein response (PERK/eIF2α), was reduced at 2–4 days (P < 0.05, Fig. 3a), followed by a recovery at 10 days to a level that was slightly, but not significantly (P = 0.19), higher than the sham level. Phosphorylated PERK (Thr980) was not detectable at 2–10 days (Fig. 3a), but at 6w a faint band was seen (P = 0.007 vs. sham, Fig. 3a). Total PERK decreased in obstructed bladders (P < 0.001 for all times, Fig. 3a). Changes at the mRNA level of Atf4 (1.22-fold, Q = 0.75, P = 0.03; 1.15-fold at 6w, Q = 1.5, P = 0.007) and Ddit3 (0.87-fold, Q = 5.4, P = 0.18; 0.86-fold at 6w, Q = 4.6, P = 0.15) downstream of PERK/eIF2α did not unambiguously support PERK activation at 10 days. We therefore concluded that at least two of the classical arms of the unfolded protein response are activated in rat bladder outlet obstruction, but with apparently distinct time-courses.

With the view to use transgenic mice, we next examined Thbs4 induction and ATF6α activation in C57BL/6J mice subjected to infravesical outlet obstruction. Both Thbs4 mRNA and protein were increased after obstruction also in this case (Fig. 3f,g). Similar to rat, Thbs4 accumulation was associated with an increased level of ATF6α-P50 (again demonstrated using two different antibodies, c.f. Supplemental Fig. 1b,c), and with increased expression of the downstream targets Pdi, Calr, Sdf2l1 and Manf (Fig. 3g). We next correlated Thbs4 induction with ATF6α cleavage and ATF6α cleavage with Pdi induction in the mouse data sets. These analyses revealed significant correlations in both cases (Fig. 3h,i).

### Outlet obstruction increases nuclear staining for ATF6α in detrusor SMCs

**Figure 4:**
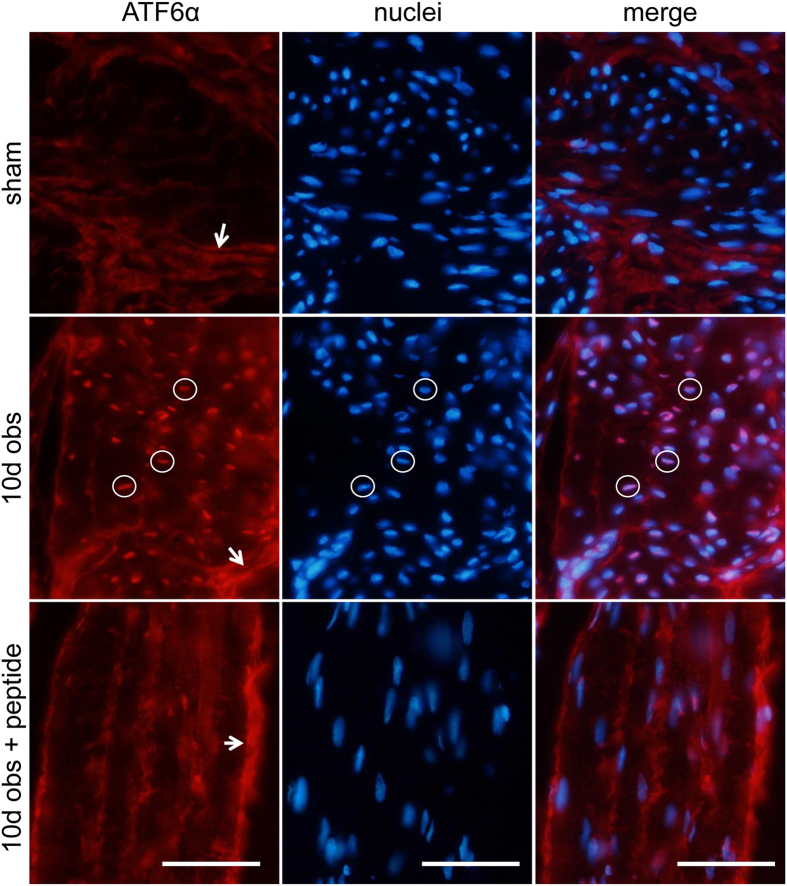
Increased nuclear immunoreactivity for ATF6α in SMCs following bladder outlet obstruction. Increased nuclear (Dapi, blue, middle column) ATF6α (red, left column) staining was observed at 10 days of outlet obstruction (middle row) compared to sham (top row). Three positive nuclei are highlighted using white circles. Incubation with blocking peptide in excess eliminated nuclear staining (bottom row), but did not affect staining of the permysium. Bars represent 50 μm.

We next examined ATF6α distribution using immunohistochemistry in bladders from sham-operated and obstructed (10 d) rats. Staining for ATF6α (red) was diffusely distributed over the bladder muscle bundles in sham-operated rats (Fig. 4, left, top row). Fluorescence was also seen in the connective tissue surrounding individual bundles (white arrows). In obstructed bladders, increased ATF6 staining of SMC nuclei was evident (white circles, left, middle row) as shown by double staining with Dapi (middle column). After pre-incubation with blocking peptide, nuclear immunoreactivity was absent, but staining of connective tissue was still present (Fig. 4, bottom panels). This argues that nuclear staining in SMCs is specific and that it increases in obstruction.

### Bladder outlet obstruction expands the ER and the secretory apparatus in SMCs

**Figure 5:**
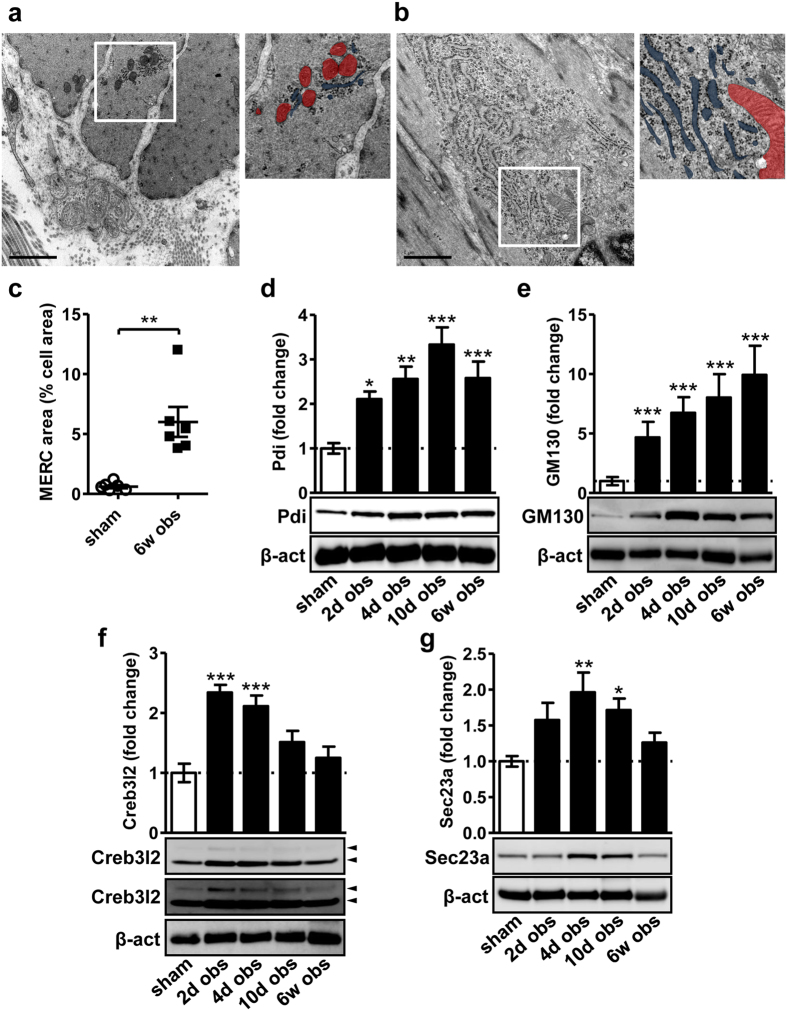
Expansion of the ER at 6 weeks of outlet obstruction. Detrusors from sham-operated rats and from rats obstructed for 6 weeks were examined using electron microscopy (**a,b**). The areas indicated by white boxes are shown at higher magnification to the right, and here the mitochondria and ER were labeled red and blue, respectively. The conglomerate of ER and mitochondria in panel b is pyramid-shaped with its base at the nucleus at the bottom right. Bars represent 1 μm. Panel c shows summarized data on such Mitochondrial ER Conglomerates (MERC) in sham-operated and obstructed bladders. Panels d through (**g**) show western blots for the ER marker Pdi, the Golgi marker GM130, Creb3l2 and Sec23a, respectively. For Creb3l2 (**f**) two different exposures are shown to highlight the weak full length band which was induced by obstruction (Creb3l2 mRNA increased by 26% at 10 days of obstruction, Q = 0, P = 0.0001, GEO accession number GSE47080). Full length and cleaved Creb3l2 (≈60 kDa) are indicated by arrowheads. The lower band was analyzed for the bar graph.

We next examined the ER in bladder SMCs from obstructed (6 weeks) and sham-operated control rats using electron microscopy (Fig. 5a,b). Aggregates of ER (blue in the smaller panels in a and b) and mitochondria (red) were seen preferentially at the nuclear poles of SMCs. Quantitative morphometry of such Mitochondrial ER Conglomerates (“MERCs”) demonstrated a ten-fold increase in their relative area in obstructed bladders (Fig. 5c). We have previously demonstrated that mitochondrial area is unchanged at 6 weeks of outlet obstruction^32^, arguing that MERC expansion probably reflects increased ER volume. Western blotting for the ER marker Pdi showed a robust increase that was maintained throughout 6 weeks of obstruction (Fig. 5d), further supporting ER expansion. MERCs occasionally contained stacks of Golgi and, accordingly, the Golgi marker GM130 was also increased (Fig. 5e). Taken together, these analyses established expansion of the ER and Golgi apparatus in SMCs following outlet obstruction.

As SMCs lose contractility during phenotypic modulation they start to synthesize and deposit extracellular matrix proteins and this requires vesicle trafficking from the ER via Golgi and onwards to the extracellular space. Vesicle budding from the ER and transport to Golgi is controlled by another ER-resident effector called Creb3l2. Cleaved Creb3l2 increases transcription of Sec23a, which directly promotes vesicle trafficking^33^. We found increased levels of Creb3l2 (both cleaved and full length) at 2–4 days and of Sec23a at 4–10 days (Fig. 5f,g), respectively. Altogether, our results thus show that bladder outlet obstruction expands the secretory apparatus in detrusor SMCs via different ER-resident effectors.

### Obstruction causes a transient reduction of contractile differentiation

**Figure 6:**
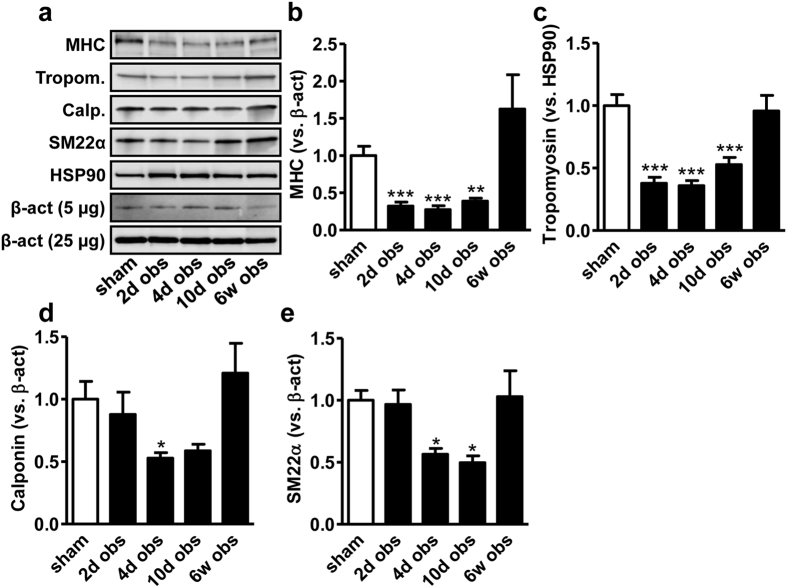
Coordinated repression of contractile marker in rat bladder outlet obstruction. Panel a shows stack of blots for myosin heavy chain (MHC), tropomyosin (Tropom.) calponin (Calp.) and SM22α. 5 or 25 μg of protein was loaded and hence two different β-actin loading controls are shown. Panels b through (**e**) show summarized data from the western blots in a. Normalization is to β-actin throughout, except for tropomyosin where the β-actin blot failed and Hsp90 was used instead.

We predicted that contractile markers would be reduced in conjunction with the elevation of synthetic markers. In support of this hypothesis, we found that all contractile markers examined were reduced at 4–10 days (Fig. 6a through e) when the synthetic markers peaked (c.f. Fig. 3d,e). This suggested coordinated reciprocal regulation of contractile and synthetic SMC markers. Without exception, the expression of contractile markers then recovered between 10 days and 6 weeks of outlet obstruction, arguing that loss of contractile differentiation is transient.

### ATF6α is activated by cell culture and is not further activated by Thbs4 transduction

**Figure 7:**
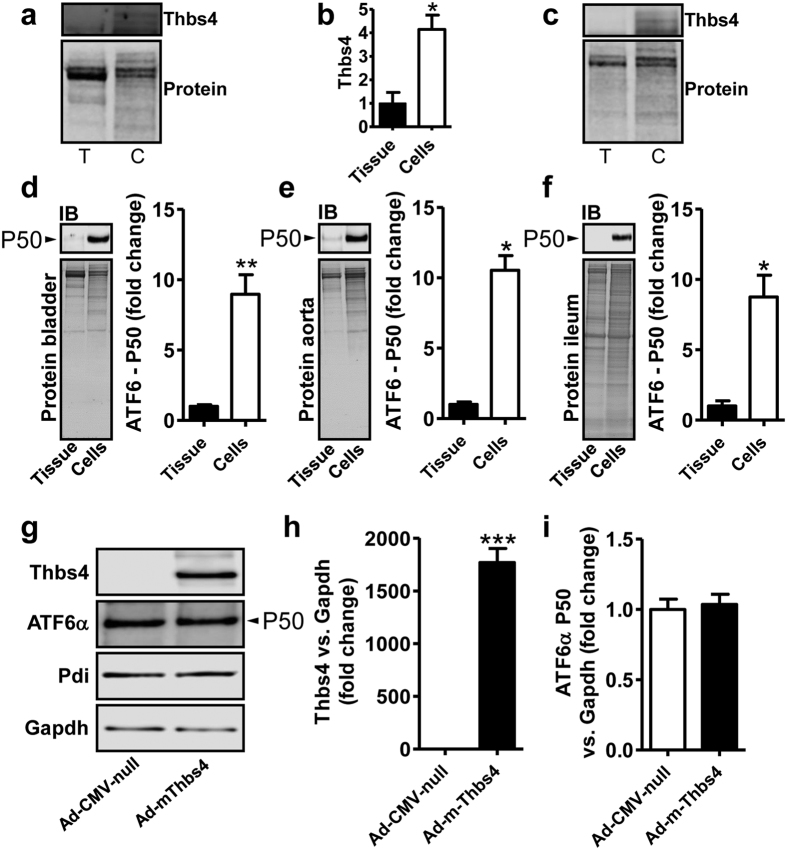
Isolation and culture of detrusor SMCs activates Thbs4/ATF6α and overexpression of Thbs4 fails to cause further ATF6α activation. Bladder, aorta and ileum were harvested from C57BL/6J mice and divided into equal halves. One half was immediately frozen. Cells were isolated and cultured from the other half. Protein lysates were made and expression in tissue (T) and cells (C) was compared by western blotting. Because none of the loading controls were stable between cells and tissue we used proteins remaining on the gels for normalization in these experiments. Panel a shows blotting for bladder Thbs4 (top) and proteins on the gel (bottom). Panel b shows summarized data from experiments in (**a**). Panel c shows blotting for Thbs4 in ileum. Panels d through (**f**) show ATF6α-P50 in tissue and cells from bladder, aorta and ileum respectively. Close to 10-fold ATF6α activation was seen in cells compared to tissue in each case. Panels g through (**i**) show the effect of adenoviral overexpression of Thbs4 in mouse bladder SMCs. Western blotting (**g**) showed close to 2000-fold overexpression of Thbs4 (**h**), but no further increase of the ATF6α-P50 level (**i**).

With the aim to test causality between Thbs4 induction and ATF6α cleavage we next isolated and cultured mouse SMCs from bladder, aorta and ileum. Consistent with the widely accepted view that SMCs undergo phenotypic modulation in culture we found an increased level of Thbs4 in cultured bladder SMCs compared to bladder tissue (compare the lanes labeled T = tissue and C = cells in Fig. 7a, and the summarized data for bladder in 7b). Similar results were obtained in ileum (Fig. 7c) and aorta (not shown). We also tested if cell dissociation and culture was associated with increased ATF6α-P50 levels and found that ATF6α-P50 was increased in cultured visceral and arterial SMCs (Fig. 7d through f). These findings presented a problem because high basal Thbs4 expression and ATF6α activation in cultured cells may preclude detection of additional ATF6α activation upon overexpression of Thbs4. Indeed, >1000-fold overexpression of Thbs4 in cultured mouse bladder SMCs (Fig. 7g,h) caused no further increase of ATF6α-P50 (Fig. 7g,i) and little change of Pdi (Fig. 7g). This ruled out overexpression of Thbs4 in cultured SMCs as a strategy to examine whether Thbs4 induction leads to ATF6α activation, and instead argued that dissociation and culture of SMCs may be sufficient for Thbs4 induction and ATF6α activation.

### ATF6α transduction induces Pdi and expands the ER

**Figure 8:**
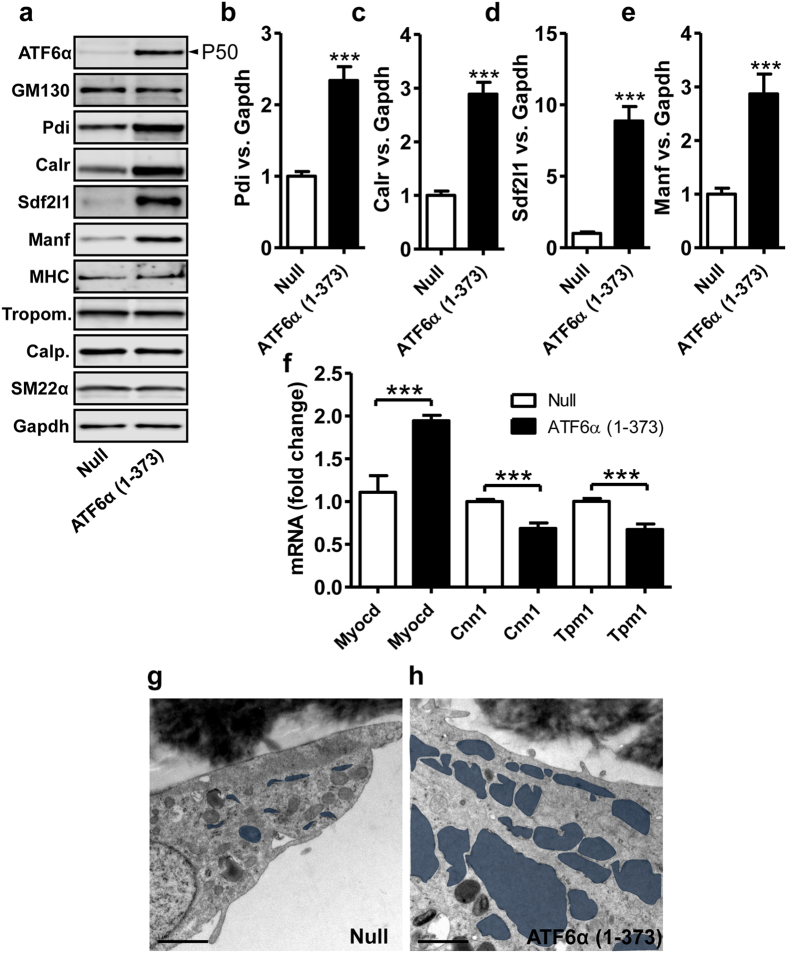
Overexpression of constitutively active ATF6α in cultured mouse detrusor cells increases Pdi, Calr, Sdf2l1 and Manf. Cells were transduced with Ad-CMV-null or Ad-CMV-ATF6α (amino acids 1–373) and ATF6α target proteins as well as smooth muscle marker proteins were examined by western blotting (**a**). Panels b through (**e**) show summarized data for Pdi, Calr, Sdf2l1 and Manf. No effect was seen on any of the contractile markers (summarized data in Supplemental Fig. 3a–d) or on GM130. Panel f shows qRT-PCR for myocardin and two of the contractile markers (calponin: Cnn1, tropomyosin: Tpm1). Panels g and h show bladder smooth muscle cells cultured on membrane inserts and transduced with Ad-CMV-null and Ad-CMV-ATF6α, respectively. ER cisterns are labeled blue and bars represent 1 μm.

We next examined if viral transduction of constitutively active ATF6α (amino acids 1–373) would result in target gene activation and ER expansion. Transduction of ATF6α increased the ATF6α protein level (Fig. 8a, see also Supplemental Fig. 1d) and moreover increased Pdi, Calr, Sdf2l1 and Manf (Fig. 8a and summarized data in b through e). Transduction did not affect the Golgi marker GM130 or any of the SMC differentiation markers at the protein level (Fig. 8a, and summarized data in Supplemental Fig. 3a through d). The mRNA levels of calponin (*Cnn1*) and tropomyosin (*Tpm1*) were however slightly repressed, and the myocardin mRNA level was increased (Fig. 8f). To test if ATF6α overexpression also expands the ER, we transduced SMCs with ATF6α or control adenovirus and examined cells by electron microscopy (Fig. 8g,h). ER cisterns were observed in 20 out of 52 micrographs in control cells and in 55 out of 86 micrographs in ATF6α-transduced cells (P = 0.003 by χ^2^-statistics). These findings show that ATF6α activation leads to target gene expression and ER expansion in bladder SMCs.

### KO of Thbs4 does not affect bladder growth, induction of ER stress genes or phenotypic modulation

**Figure 9:**
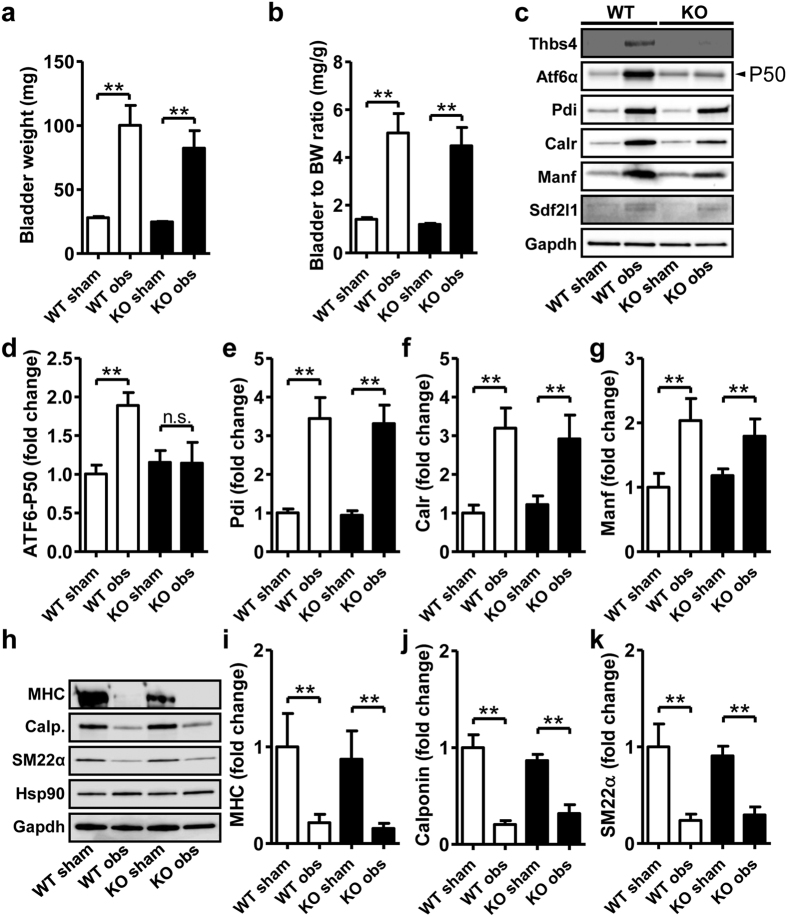
Knockout of Thbs4 does not affect growth, induction of ER stress genes or loss of contractile differentiation in the obstructed urinary bladder. Thbs4 knockout (KO) mice and littermate wild types (WT) were subjected to outlet obstruction, and bladders were harvested at 7 days. Absolute bladder weights in WT and KO mice were not different either before or following obstruction (**a**). Bladder to body weight ratios were also similar between genotypes, irrespective of obstruction (**b**). Panel c shows western blots for Thbs4, ATF6α-P50 and the ER stress markers Pdi, Calr, Manf and Sdf2l1. ATF6α-P50 formation was reduced in the obstructed KO bladder (**c,d**) but induction of Pdi, Calr and Manf was not affected (panel c and summarized data in (**e**) through (**g**)). We also examined contractile markers by western blotting (**h**), and myosin heavy chain, calponin and SM22α were similarly repressed in obstructed WT compared to obstructed KO bladders ((**i**) through (**k**)).

Using global Thbs4 knockout (KO) mice we next sought to establish a role of Thbs4 in bladder growth and ATF6α activation following outlet obstruction. Initial qRT-PCR experiments confirmed sizeable induction of Thbs4 in obstructed wild type (WT) mice (15.5 ± 4.8-fold vs. unobstructed WT mice, n = 6, P < 0.001), but not in obstructed KO mice (1.2 ± 0.3-fold vs. unobstructed KO mice, n = 6, P > 0.05) as expected. Despite lack of Thbs4 induction in KO mice, both absolute (Fig. 9a) and relative (Fig. 9b) bladder weight gains on obstruction were similar. Western blotting showed lack of Thbs4 protein accumulation, a curtailed ATF6α activation (again using two different antibodies, compare Fig. 9c and Supplemental Fig. 1c), but similar induction of Pdi, Calr, Manf and Sdf2l1 in KO compared to WT bladders (Fig. 9c and summarized data in panels d through g). We also examined the loss of contractile markers and found that repression of myosin heavy chain, calponin and SM22α were similar in WT and KO bladders (Fig. 9h and summarized data in panels i through k). This argued that while Thbs4 induction contributes to ATF6α activation in the obstructed bladder, it is not critical for elevated expression of ER stress proteins or for the loss of contractile markers.

To examine if reduced ATF6α activation was compensated for by other sensors of the unfolded protein response, we measured activation of XBP1, Creb3l2 and ATF4 in control and obstructed WT and KO bladders. Similar to rat, mouse obstruction increased the spliced 54 kDa form of XBP1 (Supplemental Fig. 2b,c). Obstruction-induced XBP1 activation tended to be greater in Thbs4 KO bladders than in obstructed WT bladders (Supplemental Fig. 2c), but this difference did not reach the level of statistical significance (P = 0.15). Activation and induction of Creb3l2 and of ATF4 by obstruction was similar between genotypes (Supplemental Fig. 2b,d,e).

## Discussion

Here, we demonstrate Thbs4 induction and ATF6α activation in bladder outlet obstruction in association with elevated expression of ER stress markers and reduced expression of contractile proteins. We moreover show that ATF6α is activated when SMCs are dissociated and cultured *in vitro* and that transduction of constitutively active ATF6α expands the ER and induces the same battery of ER stress proteins that are elevated in obstruction. However, knockout of Thbs4 neither influenced the obstruction-induced repression of SMC differentiation markers nor the induction of ER stress markers, even if activation of ATF6α was reduced. Several sensors of the unfolded protein response are however responding to outlet obstruction, suggesting a safeguard mechanism to maintain folding control when one signaling arm is selectively perturbed. Our findings therefore indicate that reciprocal regulation of synthetic and contractile markers is a key feature of bladder outlet obstruction, but that Thbs4 induction is redundant in both regards.

It has been known for decades that the endoplasmic reticulum expands as SMCs assume the synthetic phenotype^6^, but this aspect of phenotypic modulation has remained unexplained, contrasting with the wealth of knowledge that has accumulated over recent years on factors that control contractile differentiation^8^,^15^. Here we provide correlative support for the view that ATF6α may contribute to ER expansion in smooth muscle cells undergoing phenotypic modulation. We also demonstrate reduced ATF6α activation in Thbs4-defiencent bladders. A potential caveat is an altered time-course of ATF6α activation in Thbs4 knockout bladders, but if one assumes that integrated ATF6α activation is reduced, this must be compensated for by other ER stress pathways. Myocardin family coactivators do not reduce ER size themselves^34^, but XBP1 is a transcription factor activated by ER stress that has been firmly linked to ER biogenesis^35^,^36^. A candidate mechanism to compensate for reduced ATF6α activation in obstructed Thbs4-deficient bladders is therefore enhanced XBP1 activation. XBP1 activation was indeed higher, albeit not significantly, in obstructed knockout bladders. In this regard it is interesting to note that several of the ATF6α targets studied here are also targets of XBP1, including Pdi and Manf. We therefore propose that ATF6α and XBP1 readily substitute for each other, such that both pathways must be genetically or pharmacologically perturbed to cause a pathologically meaningful impact in the context of bladder hypertrophy. Alternatively, XBP1 is the transcription factor responsible for ER expansion even if ATF6α is fully capable of doing the same thing.

Based on TFBS analysis and western blotting, outlet obstruction was found to activate ATF6α and XBP1 among the classical ER stress signaling arms (ATF6α, IRE1/XBP1 and PERK/eIF2α), but our findings provide further evidence for ER stress by showing robust induction/activation of Creb3l2 (also known as BBF2H7 or SCIRR69). Similar to ATF6α, Creb3l2 is an ER resident protein that is cleaved in response to ER stress, allowing for translocation to the nucleus where it drives transcription^37^. On the basis of the finding that Creb3l2 is not only processed but also induced by ER stressors it was speculated that Creb3l2 may be preferentially involved in the late phase of ER stress signaling^37^. This speculation is not supported by our data in obstructed bladders because Creb3l2 was activated prior to ATF6α, but comparison of time-courses between the different experiments in our study should be made with caution. Work has shown that Creb3-like transcription factors regulate protein secretion^38^. Targets in the secretory pathway include GM130 (Golga2) and Sec23a^33^,^38^, both of which were increased at the protein level following outlet obstruction. GM130 is a Golgi marker and, similar to Sec23a, it is needed for ER to Golgi trafficking of proteins destined for secretion, including collagens^33^,^39^. We have demonstrated increased synthesis of a large number of extracellular matrix molecules in bladder outlet obstruction^27^,^32^. Hence, increased matrix synthesis may be the trigger of the unfolded protein response in this model, and enhanced secretion may be the critical effector mechanism.

We demonstrate a reciprocal relationship between synthetic and contractile gene expression in the detrusor following outlet obstruction. The prior finding that ATF6α can bind to SRF^20^, and thus potentially compete with myocardin family coactivators to favor the synthetic phenotype, suggested a mechanism to explain a tight coupling between loss of contractile markers and increased ER/Golgi volume. Several findings argue that it may not be so simple. For example, overexpression of constitutively active ATF6α increased all of the ER stress proteins (Pdi, Calr, Sdf2l1 and Manf), yet leaved the contractile protein levels unchanged. Moreover, and in agreement with recent work by Wang *et al.*^21^, ATF6α actually increased the mRNA level of myocardin. Genetic ablation of Thbs4, finally, impaired ATF6α activation, but did not affect either induction of ER stress proteins or repression of contractile proteins. Some indirect support for the idea that ATF6α competes with myocardin for binding to SRF was our finding that two of the contractile markers were reduced at the mRNA level following ATF6α transduction. If this has an impact on protein expression and function at later times needs to be addressed using ATF6α knockout mice. The most reasonable interpretation at present is that competition between myocardin and ATF6α for binding to SRF is offset by the simultaneous induction of myocardin, such that the impact on contractile markers is dampened.

Support for the idea that outlet obstruction affects contractile differentiation in the urinary bladder has been published^9^,^29^,^30^, but prior studies have typically considered only one marker and concentrated on a single time point. Here we provide time course data for a handful of contractile markers, demonstrating the involvement of a coordinated transcriptional program. An unexpected finding was that phenotypic modulation was transient, recovering completely at 6 weeks, despite maintained obstruction. The mechanism of this transient loss of contractile differentiation remains to be fully elucidated, but available microarray data at 10 days^27^ (GSE47080) does not support altered expression of myocardin or Mkl1 (Myocd: 1.0-fold vs. sham, Mkl1: 0.97-fold vs. sham), of SRF (1.1-fold vs. sham), or of Klf4 (0.95-fold vs. sham). We cannot rule out repression of key drivers of contractile differentiation at earlier times, but available evidence favors some other mechanism. Activated ternary complex factors compete with myocardin family coactivators for binding to SRF and control genes of relevance for growth^11^. Ternary complex factors are activated by ERK1/2^40^, and we have demonstrated transient ERK1/2 phosphorylation in rat bladder outlet obstruction with a peak at 4 days^27^, coincident with the highest growth rate. Maximal ERK1/2 activation and growth therefore matches the apparent nadir of contractile marker expression, so it is plausible that an altered balance of ternary complex factors and myocardin is responsible.

SMCs isolated from their tissue context and put into culture represents a well-established paradigm of phenotypic modulation, known to also cause loss of contractile markers in detrusor SMCs^41^. Our work on cultured SMCs demonstrated ATF6α activation in cells compared to intact tissue. This was seen using arterial, intestinal and bladder smooth muscles and therefore appears to represent a general phenomenon. Thbs4 may contribute to ATF6α activation in this situation because it was increased, potentially explaining our failure to activate ATF6α by Thbs4 overexpression, but additional mechanisms are likely involved. Recent work demonstrated elevated expression of Thbs1 in cultured SMCs versus intact aorta^42^, and Thbs1, similar to Thbs4, is capable of activating ATF6α^23^. The evidence for induction of Thbs1 in the obstructed urinary bladder *in vivo* seems however to be limited as shown by our microarray data (GSE47080).

To summarize, this study provides several insights into phenotypic modulation of detrusor SMCs following outlet obstruction and into the accompanying regulation of the protein secretory apparatus. ATF6α and XBP1-dependent ER stress signaling and attendant induction of target genes is a signature feature of obstruction, as is the coordinate but transient repression of contractile markers. Creb3l2 acts in parallel with ATF6α and XBP1 to orchestrate increased secretion. Knockout of Thbs4, finally, mitigates ATF6α activation, but has limited effects on ER stress markers or on contractile differentiation.

## Methods

### Partial bladder outlet obstruction in rats and mice

The experimental protocols were approved by the Malmö/Lund Ethical Committee for animal experiments (permit number M46-13 and M104-15) and all experiments were carried out in accordance with those protocols, with national guidelines, and with the European Communities’ Council Directive 86/609/EEC. Female Sprague-Dawley (200 g) rats were anesthetized with intramuscular injection of ketamine (Ketalar, 100 mg/kg, Parke-Davis) and xylazin (Rompun, 15 mg/kg, Bayer AG). The urethra was reached through an abdominal incision and a partial obstruction was obtained by tying 4-0 Prolene over the urethra and a 1 mm metal spacer^27^. After removal of the spacer, abdominal tissues were sutured in two layers. Rats obstructed for 2, 4 and 10 days, and 6 weeks, respectively, were compared with sham-operated controls.

Mice heterozygous for a knockout allele of thrombospondin-4 (Thbs4) were purchased from the Jackson Laboratory (B6.129P2-Thbs4tm1Dgen/J) and bred in-house to obtain wild type (Thbs4^+/+^) and knockout (Thbs4^−/−^) mice. Mice were genotyped at 4 weeks using Jumpstart Red Tac reaction mix (Sigma). Primers and cycling protocol were as recommended by Jackson. Female knockouts and their littermate controls were anaesthetized using isoflurane (2%) and a standardized obstruction was obtained as above, but using instead a 0.4 mm spacer and 6-0 Prolene. Sham-operated mice were used as controls. In experiments on mice, we limited our study to 7 days, an obstruction period that was well tolerated, because mortality was seen only after long-term obstruction. In the first training experiments on mice (Fig. 3f–i) we used wild type C57BL/6 mice. Rats and mice were euthanized using increasing CO_2_, and bladders for biochemical analysis were rapidly excised, emptied and snap frozen in liquid N_2_.

### Transcription factor binding site analysis

RNA was isolated and global gene expression analyzed by microarrays (Affymetrix). The microarray data (Figs 1a and 3d and Supplemental Figs 1e,f and 2a) is accessible at the Gene Expression Omnibus via GSE47080. In addition to sham, 10 d obstruction and 6w obstruction, this data set includes a group of rats that was obstructed for 6 weeks and then de-obstructed for another 10 days to test reversibility. Transcription factor binding site analysis indirectly assesses the impact of transcription factors in a microarray experiment. Systematic motif retrieval is used to count motifs in the promoters of differentially expressed genes and the significance of enrichment of such motifs is then calculated^43^. Our microarray data (GSE47080) and the cut-off criteria Q = 0 (by Significance Analysis of Microarrays) and either >1.2-fold or <0.8-fold change were used to generate the input gene list for the analysis. As a further test of ATF6α activation we considered the ATF6α target genes identified by Adachi *et al.*^16^ and examined whether their mean fold induction was significantly different from the hypothetical value 1 (no change) in the microarray data sets using a Wilcoxon Signed Rank Test (Supplemental Fig. 1e,f).

### Cell culture and viral transduction

SMCs were isolated from mouse bladders, from mouse intestine and from aortae by enzymatic digestion with 0.1 mg/ml elastase (Sigma) and 2 mg/ml collagenase type 2 (Worthington Biochemical Corporation) in serum free DMEM cell culture media. The SMCs were cultured in cell culture flasks containing DMEM/Ham’s F12 medium supplemented with antibiotics (penicillin and streptomycin) and 10% fetal calf serum (FCS). The flasks were placed in a water-jacketed cell incubator at 37 ^o^C with 5% CO_2_ in air. SMCs were used in passages 2–5.

To examine if increased Thbs4 expression led to ATF6α activation, smooth muscle cells from mouse urinary bladder were transduced with 50 MOI Ad-CMV-m-Thbs4 or a corresponding concentration of empty vector (Ad-CMV-Null) in DMEM/Ham’s F12 media with 10% FCS and antibiotics. After 72 hours, cells were harvested for analysis. For overexpression of ATF6α we used Ad-CMV-h-ATF6α (amino acids 1–373). All viruses were obtained from Vector Biolabs. SMCs from mouse bladders were treated with 50 MOI Ad-CMV-h-ATF6α or 50 MOI Ad-CMV-Null in growth media supplemented with 2% dialyzed FCS and antibiotics for 72 hours. Before transduction, cells were starved for 2 days. Successful adenoviral transduction was confirmed using real-time quantitative PCR and western blotting. Experiments with mouse SMCs are from 3–6 independent replicates using cells from both males and females.

### RNA extraction and qRT-PCR

After washing with ice-cold phosphate buffered saline (PBS, Biochrom), SMCs and frozen bladders were lysed in Qiazol (Qiagen). The Qiagen miRNeasy mini kit (Qiagen #217004) was used and RNA was extracted in a QIAcube (Qiagen) according to manufacturer’s instructions. The concentration and quality of isolated RNA was determined using the Nanodrop spectrophotometer (Thermo Scientific). PCR reactions were performed using the Quantifast SYBR Green RT-PCR kit (Qiagen, #204156) and Quantitect (Qiagen) primer assays for Thbs4, Atf6, Myocd, Cnn1 and Tpm1, respectively. Primer sequences are proprietary information of Qiagen. Expression of mRNAs was assessed using a real time thermal cycler (StepOnePlus™, Applied Biosystems) and 18S was used as housekeeping gene^27^.

### Western blotting

Frozen bladders were crushed in a metal press pre-cooled in liquid N_2_, and rapidly transferred to Laemmli Sample buffer (60 mM Tris-Hcl, pH 6.8, 10% glycerol, 2% SDS) to which phosphatase and protease inhibitors (Bio-Rad) had been added. Following dissolution of the tissue, the protein concentration was determined using the Biorad DC protein assay and adjusted to 1 μg/μl. Bromophenol blue (0.01%) and β-mercaptoethanol (5%) were then added. Cells were washed twice with PBS and then lysed in 50–70 μl of Laemmli sample buffer. 5–25 μg protein was loaded per lane on pre-cast TGX Criterion gels (AnyK_D_ or 4–15%, Bio-Rad). Precision Plus (Kaleidoscope, Bio-Rad) protein standard was loaded in at least one lane. After protein separation and semi-dry transfer to nitrocellulose for 10–20 min in the Trans-Blot Turbo system, we cut the membranes horizontally to allow for blotting of multiple targets using the following antibodies: thrombospondin-4 (Thbs4, sc-7657-R and sc-390734, Santa Cruz Biotechnology, AF2390, R&D systems and PA5-34386, Thermo Scientific), ATF6α (Atf6, ab11909 (a.k.a. 70B1413, 1:250, for rat and mouse), abcam, NBP1-40256 (5 mg/ml, rat and mouse), Novus, and ab37149 (for human P50), abcam), XBP1 (ab198999, abcam), P-eIF2α (Ser51, #9721, Cell Signaling Technology), eIF2α (#9722, Cell Signaling Technology), phospho- and total PERK (Cell Signalling #3179, #3192), Hsp90 (610418, BD Transduction Laboratories), β-actin (A5441, Sigma), Calr (#12238, Cell Signaling Technology), Manf (ab67271, abcam), Sdf2l1 (HPA005638, Sigma), Pdi (#3501, Cell S ignaling Technology), GM130 (NB110-57012, Novus Biologicals), Creb3l2 (ab181490, abcam), Sec23a (#8162, Cell Signaling Technology), ATF4 (#11815, Cell Signaling Technology), smooth muscle myosin heavy chain (Myh11 or MHC, ab53219, abcam), tropomyosin (Tpm1, #3910, Cell Signaling Technology), calponin (Cnn, ab46794, abcam), SM22α (Tagln, ab14106, abcam) and Gapdh (MAB374, Millipore). β-actin and Hsp90 were used as loading controls in rats as their expression remains stable^27^. For mouse on the other hand we noted that β-actin changed modestly but significantly with obstruction and therefore instead used Gapdh. When we compared tissue and cells, none of proteins used above as loading controls appeared stable. We therefore used Coomassie staining of proteins remaining on the gels after transfer for normalization.

### Immunostaining

Sham-operated and obstructed bladders were fixed in PBS containing 4% formaldehyde, dehydrated and embedded in paraffin. 5 μm cross-sections were dewaxed, rehydrated with descending concentrations of ethanol and rinsed in distilled water. Tissues and cells were blocked with 3% bovine serum albumin (BSA) for 2 h at room temperature and then incubated overnight at 4 °C with a rabbit Thbs4 and ATF6α antibodies (1:100 in 3% BSA), followed by blocking of endogenous peroxidase activity with 4% H_2_O_2_ in methanol for 20 minutes at room temperature. Immunoreactivity was visualized using secondary antibodies conjugated with HRP (horseradish peroxidase, Cell Signaling) or with Alexa 555 (Invitrogen, A21422) at 1:200 dilution. Nuclei were counterstained with hematoxylin (Histolab) or with DAPI (Invitrogen). 3,3´-diaminobenzidine tetrahydrochloride (DAB, Dako) and fluorescence were analyzed using an Olympus DP72 microscope equipped with a digital camera. Images were captured using Olympus CellSensDimension software.

ATF6α antibody (NBP-1-20256, Biotechne 1:100) was incubated with blocking peptide (H00022926, Biotechne) in excess (1:3) over night at 4 degrees.

### Electron microscopy

Rat bladders (six controls and six obstructed for six weeks) were taken out with their urine content, and transferred to fixative (2, 5 mM glutaraldehyde in 150 mM sodium cacodylate buffer, pH 7.4). After 30 min the bladders were cut open and transferred to new fixative for at least 2 hours. Mid-ventral sections were dissected, and post-fixed for 2 hours in 1% osmium tetroxide, block-stained in uranyl acetate, and embedded in Araldite. Thin sections were cut, grid-stained with lead citrate, and studied at 25 K using a JEOL JEM 1230 microscope. Relative cell area consisting of ER was measured using Image J software. The numbers given are based on 352–604 μm^2^ cell area per specimen.

Mouse bladder smooth muscle cells were seeded in 6-well plates with polycarbonate cell culture inserts (PIHP03050, Millipore). After 72 h of treatment with 50 MOI of Ad-h-ATF6α(1–373) or Ad-CMV-null viruses, cells were immersed in fixative followed by washing, post-fixation and processing as described^34^. Sections were cut and examined in an electron microscope (JEOL 1230, Jeol, Tokyo, Japan). For analysis of digital micrographs Image J was used (NIH, Bethesda, MD, USA).

### Statistical analyses

Means ± S.E.M. are given in all graphs. P-values were calculated using one way ANOVA followed by either Dunnett’s or Boneferroni’s multiple comparison tests. For single comparisons we used an unpaired Student’s t-test. Correlations were tested using Spearman analysis. The χ^2^-test was used to test the effect of ATF6α overexpression on the frequency of endoplasmic reticulum cisterns. P < 0.05 was considered to denote a significant difference.

## Additional Information

**How to cite this article**: Krawczyk, K. K. *et al.* Assessing the contribution of thrombospondin-4 induction and ATF6α activation to endoplasmic reticulum expansion and phenotypic modulation in bladder outlet obstruction. *Sci. Rep.* **6**, 32449; doi: 10.1038/srep32449 (2016).

## Supplementary Material

Supplementary Information
