# Supplementary material for: Assessing the contribution of thrombospondin-4 induction and ATF6α activation to endoplasmic reticulum expansion and phenotypic modulation in bladder outlet obstruction
Source: Sci Rep. 2016 Sep 1;6:32449. doi: 10.1038/srep32449 (PMC5007532; doi:10.1038/srep32449)
Supplement: Supplementary Information [file srep32449-s1.pdf]

Assessing the contribution of thrombospondin-4 induction and ATF6α activation to endoplasmic reticulum expansion and phenotypic modulation in bladder outlet obstruction

Authors: Katarzyna K. Krawczyk, Mari Ekman, Catarina Rippe, Mario Grossi, Bengt-Olof Nilsson, Sebastian Albinsson, Bengt Uvelius, Karl Swärd.

Supplemental material

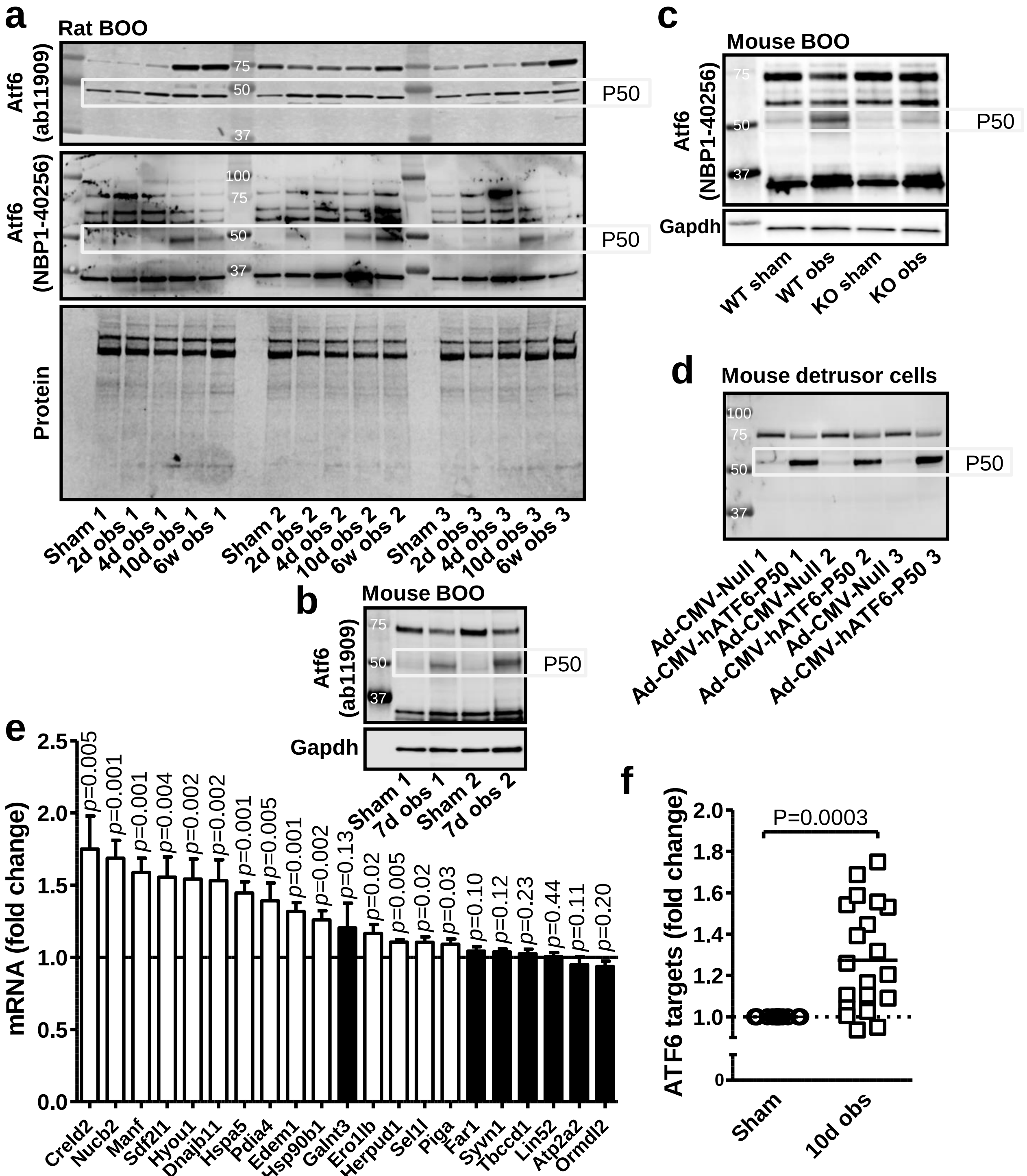

Supplemental Figure 1. Panels a through d show blots for ATF6α using different antibodies. Panel a shows rat bladder outlet obstruction (BOO), and panels b and c mouse BOO. A consistent finding is the increase of a band close to 50 kDa in obstructed bladders after 7-10 days. The numbers 1, 2, and 3 below the blots denote different experiments, but the experiments are not the same for the two antibodies as they were performed at different times. Panel d shows viral overexpression of ATF6α-P50 in mouse bladder myocytes. Note appearance of a band at the same position as endogenous P50. Loading controls for panel a (top left) and panel d are shown in Fig. 3a and Fig. 7a of the paper. In e, fold changes and raw P-values for ATF6α target genes at 10 days of rat bladder outlet obstruction are given (GEO accession number GSE47080). All white bars indicate nominally significant increases. Panel f shows a Wilcoxon signed rank test where the fold changes of ATF6α target genes were compared with the theoretical value 1 (sham).

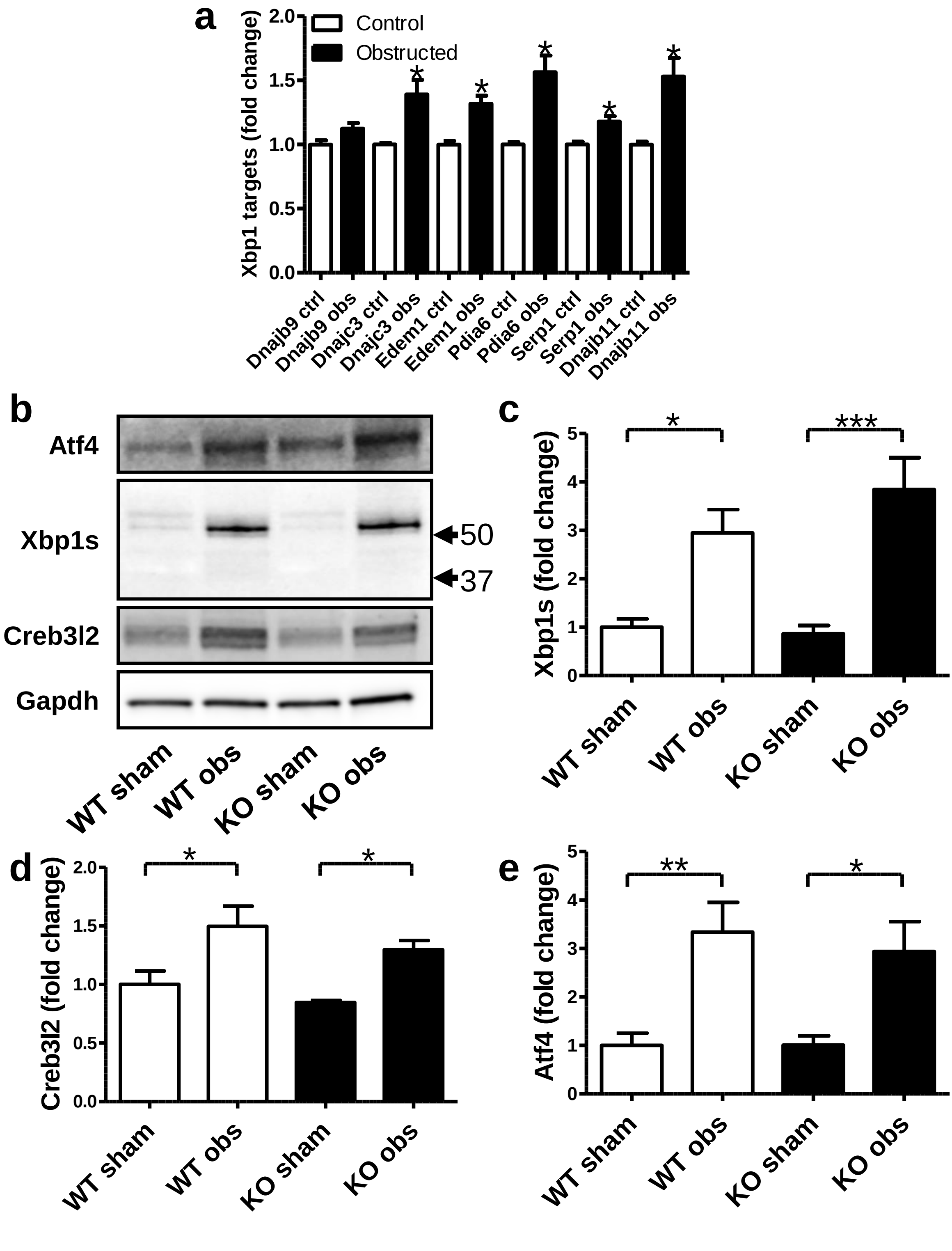

Supplemental Figure 2. Panel a shows gene targets specific for Xbp1 at 10 days of rat bladder outlet obstruction, measured by microarray analysis (GEO accession number GSE47080). Nominal P-values below 0.05 for the comparison of sham versus obstructed are indicated by *. Panel b shows western blots for mouse Atf4, spliced Xbp1 (Xbp1s) and Creb3l2 in control and obstructed wild type (WT) and Thbs4 knockout (KO) bladders. Summarized results from the blots in b (n=6) are shown in panels c through e.

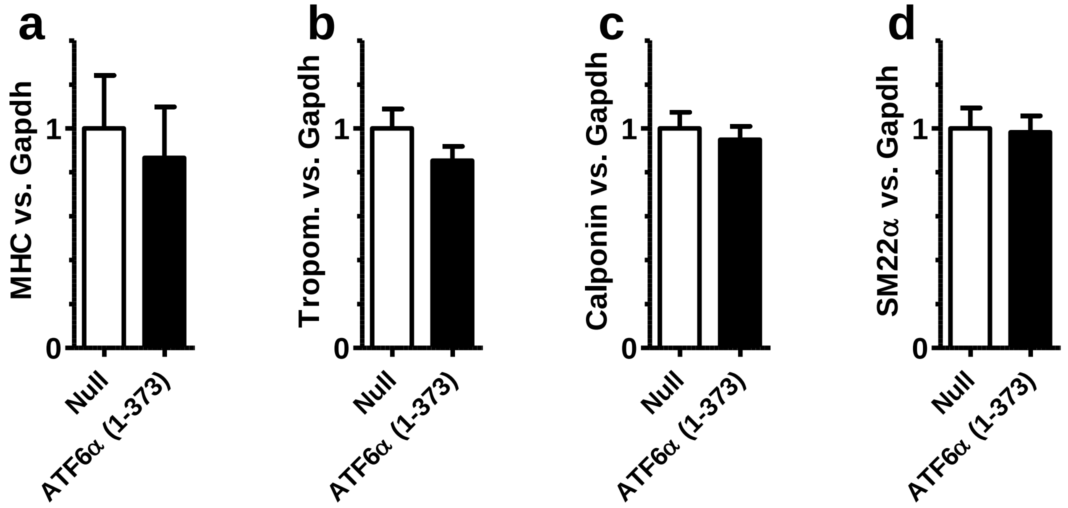

Supplemental Figure 3. Panels a through d show summarized data for smooth muscle differentiation markers after transduction with Ad-CMV-null and Ad-CMV-ATF6α (1-373), respectively. Original blots for these bar graphs are shown in the stack in Fig. 8a.
